# The Influence of Birth Weight on Cognitive Reserve and its impact on one-year Functioning in First-Episode Psychosis patients

**DOI:** 10.1192/j.eurpsy.2025.421

**Published:** 2025-08-26

**Authors:** M. F. Forte, S. Amoretti, A. Sánchez-Torres, L. Pina-Camacho, E. Vieta, C. García-Rizo

**Affiliations:** 1 Bipolar Disorders Unit,Hospital Clinic,Institute of Neurosciences, UB, IDIBAPS, CIBERSAM, Barcelona, Spain; 2 Group of Psychiatry, Mental Health and Addictions,VHIR, CIBERSAM, Barcelona, Spain, Barcelona; 3 IdiSNA, Navarra Institute for Health Research, Pamplona, Spain, Pamplona; 4 Child and Adolescent Psychiatry Department, Hospital General Universitario Gregorio Marañón,Madrid, Spain, Madrid; 5 Barcelona Clinic Schizophrenia Unit,UB, IDIBAPS, CIBERSAM, Barcelona,Spain., Barcelona, Spain

## Abstract

**Introduction:**

Cognition is key to long-term outcomes in first-episode psychosis (FEP). Obstetric complications affect working and verbal memory in schizophrenia (Amoretti *et al*. Psychol Med, 2022;52:2874-2884). Among them, birth weight (BW), an indicator of prenatal development, has been studied in relation to cognition (Krishna *et al*. Int J Geriatr Psychiatry, 2019;34:1139-1169). Higher cognitive reserve (CR) is associated with better cognitive and functional outcomes (Amoretti *et al*. Psychol Med, 2022;52:526-537). It is hypothesized that low BW impacts fetal brain development, reducing CR and later functioning (Krishna *et al*. Int Psychogeriatr, 2021;1-14)

**Objectives:**

To examine the relationship between BW and functioning one year after the onset of psychosis, mediated by CR and cognitive performance

**Methods:**

117 FEP patients and 224 healthy controls (HC) were recruited. BW was collected as a continuous variable in grams. The Functioning Assessment Short Test (FAST) assessed functioning. CR was quantified via premorbid IQ, educational attainment, and lifetime participation in leisure and social activities. A complete neurocognitive assessment was performed. Correlational analyses explored relationships between BW, CR, and cognition in FEP and HC. Serial mediation analysis (PROCESS Model 6) examined indirect effects of BW on functioning through CR and cognition

**Results:**

No significant differences were found in BW between patients and HC (p=0.719). HC had higher CR than patients (p<0.001) **(Figure 1)**. In patients, BW correlated with CR (r=0.20, p=0.027), though no correlation was found in HC (r=0.10, p=0.216) **(Figure 2)**. The mediation model confirmed that BW was associated with CR (β=0.01, p=0.015), CR was linked to verbal memory (β=2.21, p=0.001) and verbal memory with functioning (β=0.07, p=0.004). The direct and total indirect effects of BW on functioning were non-significant. Among the indirect paths, only the one involving CR and verbal memory was significant (95%CI [0.0001, 0.0016]) **(Figure 3)**.

**Image 1:**

**Figure:**
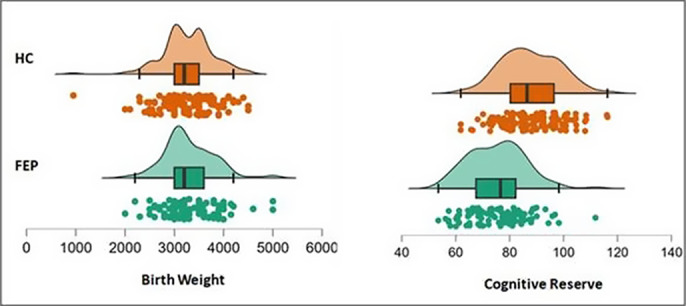

**Image 2:**

**Figure:**
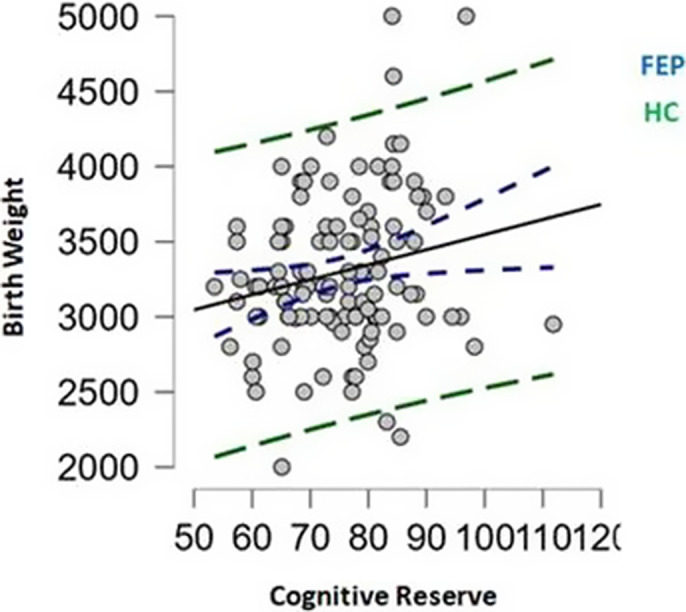

**Image 3:**

**Figure:**
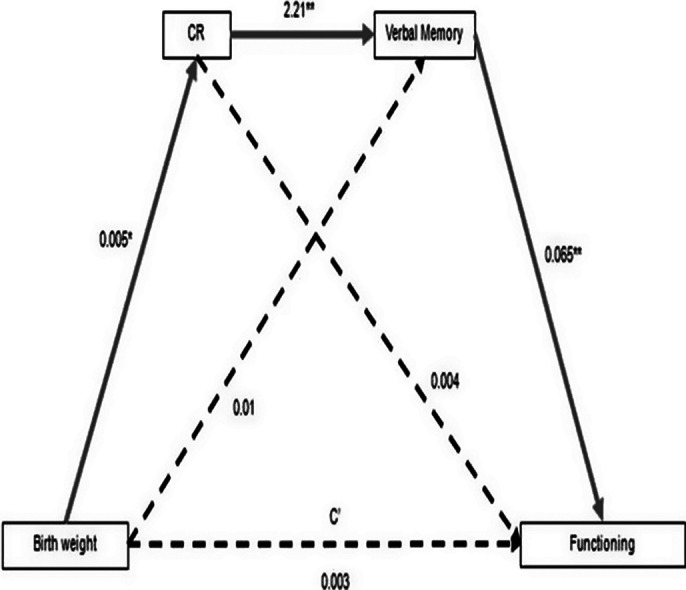

**Conclusions:**

While BW did not have a direct impact on psychosocial functioning, it does influence CR, which in turn affects verbal memory.

**Disclosure of Interest:**

None Declared

